# The Impact of Sleep Quality on Subjective Wellbeing Among Older Adults With Multimorbidity: A Moderated Mediation Model

**DOI:** 10.3389/fpsyg.2022.813775

**Published:** 2022-03-31

**Authors:** Chichen Zhang, Fang Dong, Xiao Zheng, Yaqing Xue, Shujuan Xiao, Lei Shi, Benli Xue, Jiachi Zhang, Weiyan Ou

**Affiliations:** ^1^School of Health Management, Southern Medical University, Guangzhou, China; ^2^Department of Health Management, Nanfang Hospital, Southern Medical University, Guangzhou, China; ^3^Institute of Health Management, Southern Medical University, Guangzhou, China; ^4^School of Public Health, Southern Medical University, Guangzhou, China

**Keywords:** older adults, multimorbidity, subjective wellbeing, a moderated mediation model, negative emotions

## Abstract

**Background:**

Studies have found that poor sleep quality is negatively associated with subjective wellbeing in older adults, but the mechanisms underlying are unclear. In this study, we aimed to examine the mediating role of negative emotions and the moderating role of perceived social support in the relationship between sleep quality and subjective wellbeing in older adults with multimorbidity.

**Methods:**

A multi-stage random sampling method was used to select a sample of 3,266 older adults aged 60 years and older. The Memorial University of Newfoundland Scale of Happiness (MUNSH), Pittsburgh Sleep Quality Index (PSQI), Depression Anxiety Stress Scales-21 (DASS-21), and Perceived Social Support Scale (PSSS) were used to assess subjective wellbeing, sleep quality, negative emotional states, and perceived social support, respectively. The moderated mediation models were examined using SPSS PROCESS Version 3.3 software.

**Results:**

Sleep quality had a significant direct effect on subjective wellbeing in older adults (*β* = −0.997, *t* = −11.783, *p* < 0.001). Negative emotions partially mediated the effect of sleep quality on subjective wellbeing (*ab* = −0.608, 95%CI: −0.728, −0.497). The indirect effect was moderated by perceived social support (*β* = −0.038, 95%*CI*: −0.062, −0.014, *p* < 0.001; *β* = −0.002, 95%*CI*: −0.004, −0.01, *p* = 0.008).

**Conclusion:**

Negative emotions increased the negative association between sleep quality and the subjective wellbeing of older adults with multimorbidity, and perceived social support played a moderating role. Psychological and behavioral interventions should be implemented as early as possible to promote mental health and enhance social support level of older adults with multimorbidity, and ultimately improve the subjective wellbeing of older adults.

## Introduction

The aging process does not always imply a decline, but rather, as a multidimensional process; aging outcomes appear to be different for different individuals. With the growth of age, the physiological function of older adults may gradually decline, followed by the increasing risk of chronic diseases (Fortin et al., 2012). Multimorbidity, commonly defined as the presence of two or more chronic diseases (Nunes et al., 2016). Because of population aging and the association of chronic diseases with advanced age, multimorbidity has been identified as a big challenge for patients and health systems worldwide (Seo et al., 2017). In the United States, data from the household survey section of the 1987 National Medical Expenditure Survey (NMES) show 45.0% of the general population and 88% of the population aged 65 years and above was troubled by one chronic condition or more and that over 75.0% of healthcare expenditures was related to the treatment of chronic conditions (Hoffman et al., 1996). According to the data research released by China Health and Retirement Longitudinal Study (CHARLS) in 2015, 68.81% of the people (age 45 and above) suffered from one chronic disease, while 41.5% had two or more at the same time (Cheng et al., 2019). Furthermore, previous studies have shown that multimorbidity leads to an increased risk of disability and death in older adults, and as the number of diseases increases, patients’ physical function scores and psychological composite scores tend to decrease, which seriously affects their functional status and quality of life (Yan et al., 1996). It can be seen that chronic diseases have become an important risk threatening older adults, and multimorbidity is also becoming a norm rather than the exception. Focusing on multimorbidity of the older adults is of great significance to alleviate the aging of the population.

Originated in positive psychology and health psychology, “subjective wellbeing” is an indicator of an individual’s level of wellbeing based on his or her subjective evaluation of life, including judgments of life satisfaction and feelings. Subjective wellbeing and health are closely related. Studies have shown that subjective wellbeing is an important factor influencing life expectancy in middle-aged and older adults (Iwasa et al., 2005). People with chronic diseases tend to report lower levels of wellbeing (Parks et al., 2020). Compared with older adults sicked with one chronic illness or none, those with multimorbidity not only suffer from multiple illnesses, but also have greater financial stress, which may lead to worse subjective wellbeing (Pang, 2016). Improving subjective wellbeing can promote healthy psychological, work, and social relationships of individuals, thus significantly improving their quality of life (Diener, 1984; Ryan and Deci, 2001). Thus, enhancing the subjective wellbeing is vital to a happy later life of older adults with Multimorbidity.

Currently, interest in subjective wellbeing of older adults has gained momentum. With the increase of age, the aging stereotype will continue to be internalized into the individual’s self-cognition of aging, that is, the aging self-stereotype. Aging self-stereotype has an important impact on individual physical and mental health in old age (Xu et al., 2021). Older adults’ subjective wellbeing is influenced by multiple factors, such as educational attainment, income level, health status, marital status, social class, stressful events, etc. (An et al., 2008). Sleep quality, among others, is particularly important. As older adults age, the depth and length of sleep decreases, and they become susceptible to sleep disorders (National Institutes of Health, 2005). Sleep disorder is independently and strongly associated with deterioration of subjective wellbeing (Yokoyama et al., 2008). Although not life-threatening, in the long run, it can impair the immune system of older adults, lead to physical illness and reduce subjective wellbeing (National Institutes of Health, 2005).

In addition to the research on the direct relationship between variables, some scholars are committed to the research on the interaction mechanism among variables. Socio-economic status (SES) is a complex phenomenon predicted by a broad spectrum of variables that is often conceptualized as a combination of financial, occupational, and educational influences (Winkleby et al., 1992). For example, researchers have roughly divided the intermediate mechanism of the relationship between SES and health into four categories (Huang and Yin, 2013): Material or structure mechanism, which mainly considers income-related medical and health accessibility, the quality of medical services, and the impact of exposure to harmful living and working environments; lifestyle mechanism, which mainly discusses the effects of diet, sports activities, and smoking and drinking; Social psychological mechanism, considering the influence of pressure and negative emotions; the mechanism of the interaction between the neighborhood environment of the community and the above factors (Adler and Stewart, 2010; Prus, 2010). In this study, we aimed to verify if sleep quality has an indirect impact subjective wellbeing through other factors.

Previous studies have shown that sleep disorder is associated with increased stress sensitivity and self-reported negative emotions (Guerrero and Crocq, 1994; Minkel et al., 2012). The negative effects of impaired sleep on physical and mental wellbeing of older adults have recently been recognized by health care professionals (Luyster et al., 2015). Sleep deprivation may impair the effectiveness of emotion regulation strategies, creating undesirable consequences as to emotional wellbeing (Zhang et al., 2019). Studies have found that poor sleep quality in older adults is associated with negative emotions, such as anxiety and depression (Wolkove et al., 2007). At the same time, negative emotions are strongly associated with subjective wellbeing in older adults.

Perceived social support refers to the emotional experience and satisfaction that individuals feel respected, supported, and understood from the society (Brissette et al., 2002). It might be used to interpret the mechanism of psychological or mental health problems (Brissette et al., 2002). Social support may influence one’s health behaviors through two main hypotheses. One is the direct effect (main effect) model, which proposes that social environments can help regulate health behaviors by providing resources directly. The other is the stress buffering hypothesis, which suggests that social support can provide resources to buffer the negative effect of stress and difficulties on health which finally maintain and improve an individual’s health outcomes (Lu et al., 2019). Early studies have found that perceived social support can have a significant positive effect on subjective wellbeing in older adults (Schulz and Decker, 1985; Farriol-Baroni et al., 2021).

**Figure 1:**
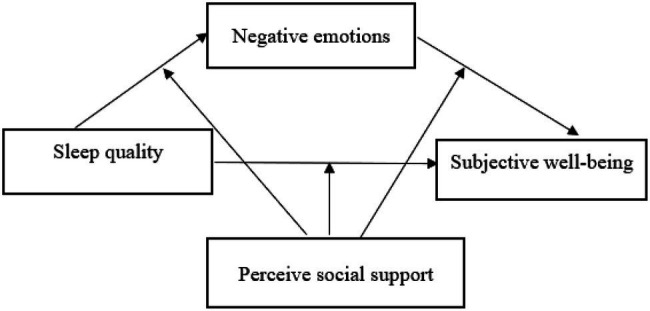
Theoretical model: a moderated mediation model.

Therefore, we hypothesized that negative emotions mediated the association between sleep quality and subjective wellbeing, and the associations were moderated by perceived social support (Figure 1).

## Materials and Methods

### Study Design and Participants

This cross-sectional study was conducted by means of a questionnaire in 11 cities in Shanxi Province, central China. Participants were selected through a multi-stage random sample. Firstly, each district (county) in every city was numbered according to the order of districts (counties) on the government’s website. Secondly, two (districts) counties in each city were selected using a random number table; two communities (administrative villages) were drawn from each district (county) in the same way. Then, we selected one or two residential communities (natural villages) from each community (administrative village), taking into account the different population size of each community (administrative village). Finally, among the residential communities (natural villages) selected, we randomly selected older adults who met the criteria in this study.

The inclusion criteria for this study were age 60 years and above; and clear cognitive and unimpeded communication skills. Those with communication difficulties were excluded. A total of 3,266 questionnaires were distributed in the study and 3,250 valid questionnaires were returned, with a valid response rate of 99.51%. All study procedures were approved by the university ethics committee. All research subjects were informed of the purpose of the investigation and signed informed consent forms. After providing consent, the participants were invited by trained investigators to respond to questionnaires, which were used to collect data.

In total, 3,250 individuals were recruited and 985 (425 male and 560 female) older adults with multimorbidity were included in the analysis, with a mean age of 71.37 (*SD* = 7.08) years old, and mean number of chronic diseases 2.80 (*SD* = 1.13).

### Measures

#### Self-Administered Demographic Characteristics Questionnaire

Demographic data of participants included gender, age, BMI, living area, education level, marital status, monthly income, empty nest status, smoking status, and drinking status, whether they have chronic diseases, and the number of chronic diseases.

Multimorbidity is a condition where a person has two or more chronic diseases at the same time (Nunes et al., 2016). Respondents were therefore considered to be suffering from multimorbidity when they answered that they had two or more chronic diseases. Although the measure of chronic diseases was made in the form of self-reporting by the respondents in the study, it required a medical record with a clear diagnosis or a doctor’s prescription certificate from a hospital in the county or above. Questionnaire on prevalence of chronic diseases includes 26 chronic diseases diagnosed by doctors, including obesity, hypertension, diabetes, coronary heart disease, stroke, arrhythmia, atherosclerosis, tuberculosis, respiratory diseases, Parkinson’s disease, chronic obstructive Pneumonia, sciatica, rheumatoid or rheumatoid arthritis, hypothyroidism, hyperthyroidism, gout, osteoporosis, hearing impairment, eye disease, hepatitis, chronic nephritis, tuberculosis, mental illness, dementia, digestive system diseases, and cancer.

#### Subjective Wellbeing

Participants’ subjective wellbeing was measured by the Memorial University of Newfoundland Scale of Happiness (MUNSH). It developed by Kozma et al., it was first applied in Newfoundland in 1980 to people aged 65–95 years in urban, rural, and older adults’ apartments. The reliability and validity of the scale were high (Martín-María et al., 2021). This scale can be used to assess the subjective wellbeing of older people in China (Zhang et al., 2021). The MUNSH contains 24 items, structured into four subscales, namely positive affect (PA) and negative affect (NA) with five items for each, positive experience (NE), and negative experience (PE) with seven items for each (Liu and Gong, 1999). The total score is equal to the positive and negative factor scores ranging from −24 to +24. For calculation purposes, the constant 24 is added, and the score range is 0–48. The higher the score, the higher subjective wellbeing. According to MUNSH evaluation criteria, the total score: ≥36 indicates high subjective wellbeing level, ≤12 indicates low subjective wellbeing level, and in between is medium subjective wellbeing level (Zhang et al., 2017). The Cronbach’s *α* for the four sub-dimensions of the MUNSH scale in this study were 0.790, 0.827, 0.746, and 0.810, with a KMO coefficient of 0.936, indicating good reliability.

#### Pittsburgh Sleep Quality Index

Sleep quality was measured by Pittsburgh Sleep Quality Index (PSQI; Buysse et al., 1989). The PSQI is a self-report questionnaire that assesses quality of sleep during the previous month. It contains 19 self-rated questions yielding seven dimensions: the total score of each dimension is accumulated as the total PQSI score, which ranges from 0 to 21 (Buysse et al., 1989), higher scores suggest poorer sleep quality (Liu et al., 2017).

#### Negative Emotions

The short-form version of the Depression Anxiety Stress Scales (DASS-21) has 21 items used to measure three negative emotional experiences with seven items for each: depression (Diener, 1984; Winkleby et al., 1992; Guerrero and Crocq, 1994; An et al., 2008; Huang and Yin, 2013; Seo et al., 2017; Cheng et al., 2019), anxiety (Hoffman et al., 1996; Iwasa et al., 2005; Yokoyama et al., 2008; Adler and Stewart, 2010; Minkel et al., 2012; Nunes et al., 2016; Pang, 2016), and stress (Yan et al., 1996; Ryan and Deci, 2001; National Institutes of Health, 2005; Prus, 2010; Fortin et al., 2012; Lu et al., 2018; Parks et al., 2020; Xu et al., 2021). A four-point scale was used (0 = not at all, 1 = partially, 2 = mostly, and 3 = fully), with higher scores indicating stronger negative emotional experience (Lu et al., 2018).

#### Perceived Social Support

Perceived Social Support Scale (PSSS) is an individual self-understanding, self-PSSS for family support, friend support, and other support, and the total score reflects the total degree of social support felt by the individual from family, friends, and others. The scale contains three dimensions: family support, friend support, and other support. A seven-level scoring method was used, with the score of all entries combined ≤36 for as low support status, ≥61 as high support status, and the rest as intermediate support status (Wei et al., 2016).

### Data Analyses

As this study used self-reported data, which may lead to a common method bias effect, Harman’s one-way analysis was used to test. Correlation analysis between variables was realized by Pearson Product–Moment Correlation. Mediation and moderation were tested using the SPSS macro program PROCESS developed by Hayes (2013). If the 95% CI of the mediation effect did not contain zero, then the effect would be significant at the 0.05 level. The macro allowed for calculating and testing direct effects, the total effect, and indirect effects. Based on the bootstrap moderating and mediation effect tests, our calculations were carried out in two steps. Model 4 examines the mediating role of negative emotions in the effect of sleep quality on subjective wellbeing. Then, Model 59 examines the moderating role of perceived social support in the relationship between sleep quality and negative emotions on subjective wellbeing. The bias-corrected percentile Bootstrap test was used to extract 10,000 repetitions to calculate the 95% CI. IBM SPSS version 26 was used for data analysis.

### Common Method Deviation Test

In Harman one-way analysis of factors test, items were subjected to unrotated principal component factor analysis. The results showed that the first factor explained 23.65% of the variance, less than 40%, indicating that the data in this study did not produce a serious common method bias.

## Results

### Descriptive Analyses

The mean, SDs, and correlations of all variables were presented in Table 1. Sleep quality (*r* = −0.387, *p* < 0.01) and negative emotions (*r* = −0.603, *p* < 0.01) were negatively correlated with subjective wellbeing. Perceived social support was positively related to subjective wellbeing (*r* = 0.492, *p* < 0.01) and negatively related to negative emotions (*r* = −0.350, *p* < 0.01). Sleep quality was positively correlated with negative emotions (*r* = 0.434, *p* < 0.01).

**Table 1:** Results of descriptive statistics and correlation analysis for each variable (*n* = 3,250).

Variables	*M*	*SD*	1	2	3	4
1 Sleep quality	5.08	3.44	1	-	-	-
2 Negative emotions	19.14	20.57	0.434^**^	1	-	-
3 Perceived social support	66.15	13.69	−0.240^**^	−0.350^**^	1	-
4 Subjective wellbeing	33.65	10.15	−0.387^**^	−0.603^**^	0.492^**^	1

*M*, mean, *SD*, standard deviation. A higher sleep quality score indicates a more serious sleep quality problem, (same below).

** *p* < 0.01.

### Mediation Effect Analysis

Control variables included gender, age, BMI, living area, educational level, marital status, monthly income, empty nest status, smoking status, and drink status. Model 4 in PROCESS program was adopted to test the mediating effect of negative emotions on sleep quality and subjective wellbeing. Table 2 presented that sleep quality had a direct predictive effect on the subjective wellbeing (*β* = −0.997, *t* = −11.783, *p* < 0.001). Sleep quality was a positive predictor of negative emotions (*β* = 2.360, *t* = 14.032, *p* < 0.001). After adding the negative emotion’ score as a mediating variable, negative emotions still had a negative prediction effect on the subjective wellbeing (*β* = −0.258, *t* = −18.604, *p* < 0.001), and sleep quality also had a negative predictive effect on the subjective wellbeing (*β* = −0.388, *t* = −4.875, *p* < 0.001). In addition, on bias-corrected percentile bootstrap analysis, Table 3 showed that the mediation effect of negative emotions on the relationship between sleep quality and subjective wellbeing was significant (*ab* = −0.608, 95% CI −0.728 to −0.497), and accounted for 61.00% of the total effect. Therefore, negative emotions partially mediated the relationship between sleep quality and subjective wellbeing.

**Table 2:** Mediating effects of negative emotions between sleep quality and subjective wellbeing.

Regression equation	Goodness of Fit	Coefficient significance			
Outcome variables	Process	*R^2^*	*F*-value	*β*-value	*t*-value
Subjective wellbeing	Sleep quality	0.195	21.380	−0.997^***^	−11.783
Negative emotions	Sleep quality	0.215	24.234	2.360^***^	14.032
Subjective wellbeing	Sleep quality	0.406	55.393	−0.388^***^	−4.875
	Negative emotions			−0.258^***^	−18.604

*** *p* < 0.001.

**Table 3:** Total effect (Fortin et al., 2012), direct effect (Nunes et al., 2016), and mediation effect (Seo et al., 2017).

Items	Effect size	*SE*	95% CI	Relative effect value
1	−0.997	0.085	(−1.163, −0.831)	
2	−0.388	0.080	(−0.545, −0.232)	39.00%
3	−0.608	0.060	(−0.728, −0.497)	61.00%

### Moderating Effect Analysis

Moderated mediation effect analysis was performed using model 59 (Wei et al., 2016) in the PROCESS program (in Tables 4–6). Table 4 showed that the effect of the interaction term of sleep quality and perceived social support on the negative emotion’s score was statistically significant (*β* = −0.038, *t* = −3.081, *p* < 0.01), which indicates that the relationship between sleep quality and subjective wellbeing was moderated by perceived social support. Additionally, the effect of the interaction term of negative emotions and perceived social support on the subjective wellbeing was statistically significant (*β* = −0.002, *t* = −2.645, *p* < 0.01). This result indicated perceived social support moderated the relationship between negative emotions and subjective wellbeing. However, the effect of the interaction term of sleep quality and perceived social support on subjective wellbeing was not statistically significant (*β* = −0.006, *t* = −1.046, *p* = 0.296).

**Table 4:** Mediating effect analysis with moderation.

Regression equation	Goodness of Fit	*R^2^*	*F*-value	*β*-value	*t*-value	*p*	*95%CI*
Outcome variables	Predictive variables						
Negative emotions		0.282	31.870^***^				
	Sleep quality			1.991	12.045	<0.001	1.666, 2.315
	Perceived social support			−0.443	−9.122	<0.001	−0.538, −0.347
	Sleep quality × Perceived social support			−0.038	−3.081	0.002	−0.062, −0.014
Subjective wellbeing		0.488	66.047^***^				
	Sleep quality			−0.291	−3.902	<0.001	−0.437, −0.145
	Negative emotions			−0.227	−16.023	<0.001	−0.255, −0.199
	Perceived social support			0.251	11.807	<0.001	0.210, 0.293
	Sleep quality × Perceived social support			−0.006	−1.046	0.296	−0.018, 0.005
	Negative emotions × Perceived social support			−0.002	−2.645	0.008	−0.004, −0.001

*** *p* < 0.001.

**Table 5:** The impact of sleep quality on negative emotions at different levels of perceived social support.

PSS	Effect size	Bootstrapped *SE*	Bootstrapped *LICI*	Bootstrapped *UICI*
*M*−*SD*	2.534	0.236	2.070	2.997
*M*	1.923	0.168	1.594	2.252
*M* + *SD*	1.503	0.234	1.044	1.962

**Table 6:** The impact of negative emotions on subjective wellbeing at different levels of perceived social support.

PSS	Effect size	Bootstrapped *SE*	Bootstrapped *LICI*	Bootstrapped *UICI*
*M*−*SD*	−0.485	0.083	−0.654	−0.333
*M*	−0.445	0.056	−0.563	−0.343
*M* + *SD*	−0.389	0.075	−0.551	−0.258

**Figure 2:**
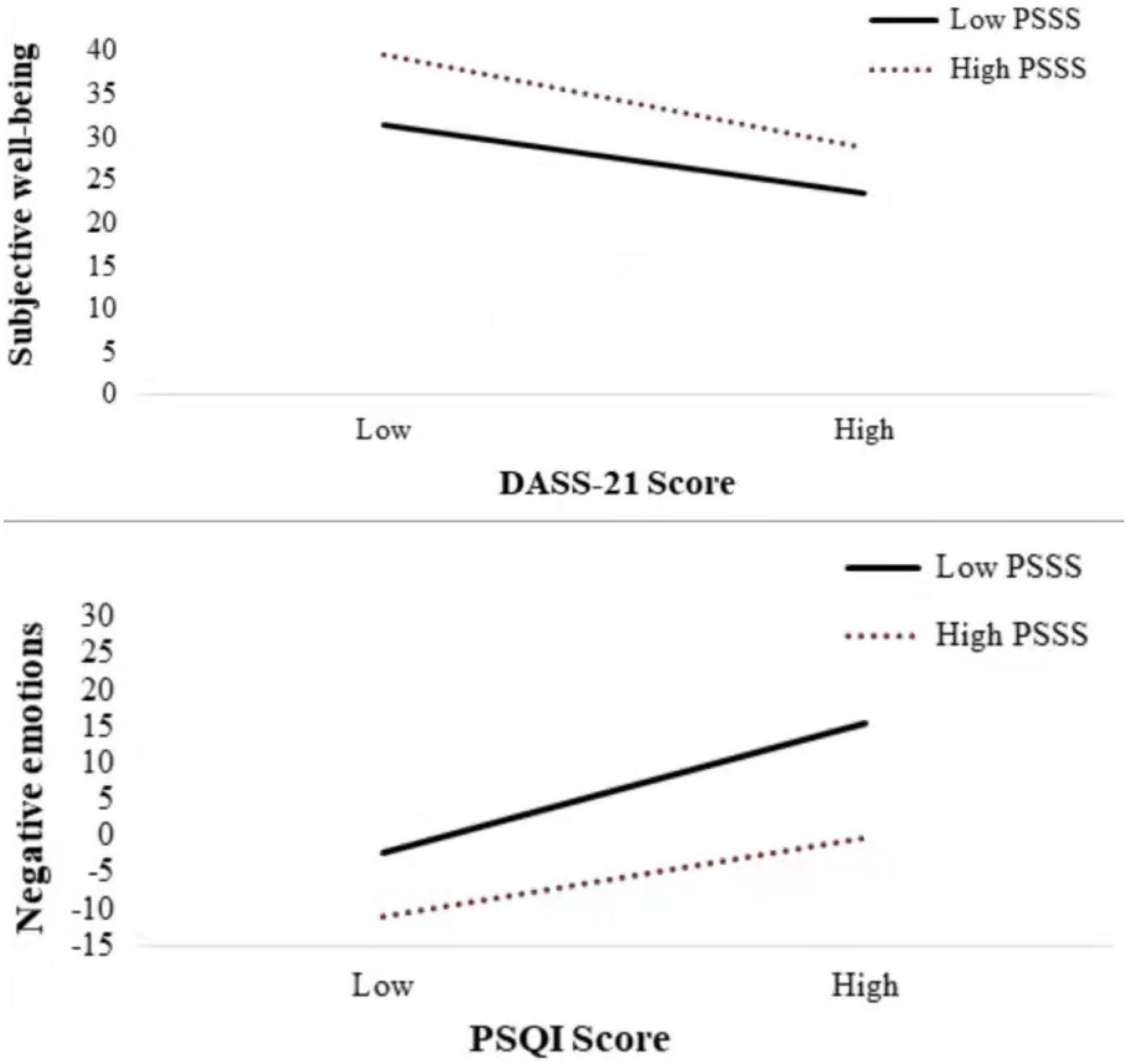
Moderation of perceived social support to sleep quality and negative emotions and moderation of perceived social support to negative emotions and subjective wellbeing.

The results of the simple slope test (see Table 5 and Figure 2) further suggest that when perceived social support is low, sleep quality is positively associated with the negative emotions (*β*_simple_ = 2.534, 95% CI 2.070–2.997). Moreover, in older adults with high perceived social support, the association between sleep quality and negative emotions was still significant (*β*_simple_ = 1.503, 95% CI 1.044–1.962). These results indicate that the relationship between sleep quality and negative emotions became weaker with an increase in perceived social support. Thus, perceived social support is a protective factor that can effectively alleviate the adverse effects of poor sleep quality on negative emotions.

The results of the simple slope test (see Table 6 and Figure 2) further suggest that when perceived social support is low, negative emotions are negatively associated with subjective wellbeing (*β*_simple_ = −0.485, 95% CI −0.654 to −0.333). Moreover, in older adults with high perceived social support, the association between negative emotions and subjective wellbeing was still significant (*β*_simple_ = −0.389, 95% CI −0.551 to −0.258). These results indicate that the relationship between negative emotions and subjective wellbeing became weaker with an increase in perceived social support. Thus, perceived social support is a protective factor that can effectively alleviate the adverse effects of negative emotions on subjective wellbeing.

## Discussion

In this study, we found that poorer sleep quality and higher negative emotions were partly responsible for poorer subjective wellbeing in older adults with multimorbidity. The indirect effect was moderated by perceived social support. The indirect effect was stronger in older adults with multimorbidity who had higher levels of perceived social support. These findings help to elucidate the potential causes of low subjective wellbeing and help to develop targeted interventions to improve subjective wellbeing in older adults with multimorbidity.

Consistent with previous studies, sleep duration is associated with subjective sleep quality and subjective wellbeing (Lemola et al., 2013). Highly optimistic people with high social support show more positive emotions and higher levels of subjective wellbeing (Quevedo and Abella, 2010). Our results showed that, both sleep quality and negative emotions were associated with subjective wellbeing in older adults with multimorbidity. Meanwhile, poor sleep quality was found to be associated with anxiety and depression in older adults (Wolkove et al., 2007). The mediation analyses also revealed a significant mediating effect of negative emotions on the association between sleep quality and subjective wellbeing. One possible explanation is that poor sleep quality has a negative impact on the physical and psychological aspects of older adults with multimorbidity. Depression and stress were also associated with poor physical health (Lee and So, 2019). Therefore, poor sleep quality may increase negative emotional problems in older adults with multimorbidity, thereby decreasing their subjective wellbeing. Subjective wellbeing of older adults is lower when sleep quality is poorer and accompanied by negative emotions. These findings suggest that the effect of sleep quality on subjective wellbeing is partially regulated by negative emotions.

In addition, perceived social support moderated the negative effect between negative emotions and subjective wellbeing. Good perceived social support promotes and maintains the individual’s mental health and reduces the generation and development of anxiety (Lu et al., 2018). Perceived social support is negatively and significantly associated with sleep quality. People with higher levels of perceived social support feel more respect, understanding, and support from others. People with a low perception of social support feel more negative emotions, such as loneliness, helplessness, and despair (Chen, 2018). More importantly, our moderating mediator analysis showed that perceived social support not only moderated the effect of sleep quality on negative emotions, but also the association between sleep quality and subjective wellbeing through negative emotions. We found that poor sleep quality can reduce an individual’s subjective wellbeing. People with poor sleep quality and lower levels of perceived social support experience more negative emotions, which further reduce subjective wellbeing. This is consistent with related research that a highly variable sleep schedule may provoke sleep problems and poor subjective wellbeing (Hayes, 2013).

Our study found that older adults with multimorbidity have lower level of subjective wellbeing. Low sleep quality and negative emotions combined can exacerbate the negative effects of subjective wellbeing. But perceived social support was found to improve sleep quality and ease negative emotions while buffering this negative effect. It is suggested that the community should take the initiative in providing monitoring services to improve sleep quality of older adults, focus on the mental health status of older adults with multimorbidity, including negative emotions, such as anxiety, depression, and stress, and encourage older adults to go outdoors, participate in public activities and communicate with friends, and promote family care for them. In addition to, we suggest that through multiple efforts to improve the subjective wellbeing of older adults with multimorbidity, thereby improving their compliance and chronic disease treatment outcomes, and laying the foundation for improving the health of the older adults.

### Limitations

It is difficult to draw from cross-sectional data causal inferences between the identified factors and subjective wellbeing. Longitudinal studies should be designed in the future to explore the causal relationship between sleep quality and subjective wellbeing.

## Conclusion

In conclusion, negative emotions increased the negative association between sleep quality and the subjective wellbeing of older adults with multimorbidity, and perceived social support played a moderating role. The increased effect was greater in older adults with multimorbidity whose perceived social support is at higher levels. Sleep quality had a significant direct effect on subjective wellbeing in older adults. The better the quality of sleep, the higher the subjective wellbeing of older adults. To improve the subjective wellbeing of older adults with multimorbidity, negative emotions interventions should be implemented as early as possible to reduce negative emotions and increase social support from family, friends, and others. Psychological and behavioral interventions should be implemented as early as possible to promote mental health and enhance social support level of older adults with multimorbidity, and ultimately improve the subjective wellbeing of older adults and achieve healthy aging.

## Data Availability Statement

The raw data supporting the conclusions of this article will be made available by the authors, without undue reservation.

## Ethics Statement

The studies involving human participants were reviewed and approved by Ethics Committee of Shanxi Medical University. The patients/participants provided their written informed consent to participate in this study. Written informed consent was obtained from the individual(s) for the publication of any potentially identifiable images or data included in this article.

## Author Contributions

CZ and FD conceived the idea. BX and JZ participated in data collection and statistical analysis. FD drafted the manuscript. LS and WO edited the paper. XZ and YX gave many valuable comments on the draft and polished it. All authors contributed to the article and approved the submitted version.

## Funding

This work was supported by the National Natural Science Foundation of China under Grant (number: 71874104); Key Laboratory Development Project for Philosophy and Social Sciences in Guangdong under Grant (number: G620369695); and National Key R&D Program of China under Grant (2020YFC2006400).

## Conflict of Interest

The authors declare that the research was conducted in the absence of any commercial or financial relationships that could be construed as a potential conflict of interest.

## Publisher’s Note

All claims expressed in this article are solely those of the authors and do not necessarily represent those of their affiliated organizations, or those of the publisher, the editors and the reviewers. Any product that may be evaluated in this article, or claim that may be made by its manufacturer, is not guaranteed or endorsed by the publisher.
